# Circ_0000190 suppresses gastric cancer progression potentially via inhibiting miR-1252/PAK3 pathway

**DOI:** 10.1186/s12935-020-01422-5

**Published:** 2020-07-29

**Authors:** Gui-Jun Wang, Tian-Yu Yu, Yan-Rong Li, Yang-Jun Liu, Bei-Bei Deng

**Affiliations:** 1 Department of General Surgery, The First Affiliated Hospital of Jinzhou Medical University, Jinzhou, 121000 China; 2 Department of Gastroenterology, The First Affiliated Hospital of Jinzhou Medical University, Jinzhou, 121000 China; 3 Department of Clinical Laboratory, The First Affiliated Hospital of Jinzhou Medical University, No. 2, Section 5, Renmin Street, Guta District, Jinzhou, 121000 Liaoning China

**Keywords:** circ_0000190, miR-1252, PAK3, Gastric cancer

## Abstract

**Background:**

Gastric cancer is a serious malignant tumor associated with aberrant circular RNAs (circRNAs) expression. In this study, we aim to investigate the role and the underlying mechanism of circ_0000190, a circRNA in gastric cancer.

**Methods:**

Circ_0000190 expression in vivo was examined in gastric cancer and adjacent normal tissues by RT-PCR. Circ_0000190 expression in gastric cancer cell lines was detected by FISH and RT-PCR. The role of the circRNA in gastric cancer cells was assessed by the analysis of cell viability, apoptosis, proliferation, cell cycle and migration. The potential effector of circ_0000190 was predicted by computational screen and validated by luciferase reporter assay. Furthermore, Mice model of human gastric cancer was established to observe the underlying mechanisms of circ_0000190.

**Results:**

Circ_0000190 was down-regulated in gastric cancer tissues and cells, with a major location in cytoplasm. Circ_0000190 inhibited gastric cancer cell viability, proliferation and migration, and induced apoptosis and cell cycle arrest by regulating the expression of capase-3, p27 and cyclin D. In addition, the circRNA was validated as a sponge of miR-1252, which directly targeted PAK3. The effects of circ_0000190 on the cellular processes were blocked by miR-1252 mimics, which could be rescued after further overexpression of PAK3.

**Conclusions:**

Circ_0000190 suppresses gastric cancer progression potentially via inhibiting miR-1252/PAK3 pathway, employing circ_0000190 might be a promising therapeutic strategy for the treatment of gastric cancer.

## Background

Gastric cancer is the fifth incidence and third leading cause of death among different cancers worldwide, which is mostly occurred in Eastern Asia, Central and South America, as well as Central and Eastern Europe [1, 2]. It was reported that the rate of new cases and deaths of gastric cancer are 5.7% and 8.2% respectively in 36 cancers in 2018 [2]. With the improvement of nutrition intake, decreased prevalence of *Helicobacter pylori* infection, and early diagnosis, the incidence of gastric cancer steadily decreased and the survival trend increased over the past few years [3]. However, limited benefit was obtained from various therapies, despite of newly developed therapeutic strategies [4]. Furthermore, patients with gastric cancer have poor outcome marked by a low rate of 5-year survival, which might be due to the lack of high specificity and high sensitivity targets [5].

Circular RNAs (circRNAs) are a newly identified family of non-coding RNAs that characterized by covalently closed loops without either 5′-3′ polarities or polyadenylated tails [6]. Growing studies have demonstrated that critical involvement of circRNAs in various types of cancers, including gastric cancer, hepatocellular carcinoma, lung and prostate cancers. Bian et al. reported that circ_103809 decreased the proliferation and metastasis of colorectal cancer cells [7]. Circ_100876 has been shown to promote growth and invasion of breast cancer cells, and suggested to be a prognostic biomarker of breast cancer [8]. Additionally, a recent study reported that an increase in circ_104916 induced cell cycle arrest, cell apoptosis and epithelial-mesenchymal transition in colon cancers [9]. In gastric cancer, circ_0000190 was noted to be down-regulated and suggested as a novel diagnostic biomarker [10]. Therefore, we hypothesized that circ_0000190 might be associated with the progress of gastric cancer.

MicroRNAs (miRNAs) are a highly conserved class of noncoding small RNAs, which recognized to act as tumor suppressors and are closely involved in the progress of many human cancers, including gastric cancer [11, 12]. The functions of CircRNAs associated with various biological process were majorly ascribed to sponging microRNAs. Recent years, a variety of miRNAs have been reported to participate in gastric cancer progression by negatively mediating the role of CircRNAs. For example, miR-296-5p was sponged by circPSMC3 and inhibiting its anti-tumorigenesis [13]. MiR-149-5p, a target of CircNRIP1, exerted protective role by blocking the malignant behavior of the CircRNA [14]. On the other hand, a report by Feng et al. showed that miR-767-5p promoted the progression of multiple myeloma and was directly targeted by circ_0000190 [15]. To some extent, this supports our hypothesis of the involvement of the CircRNA in gastric cancer.

P21-activated kinases (PAKs), a family serine/threonine kinases associated with Rac/Cdc42, are major downstream effectors of the small Rho GTPases that play an important role in a variety of cellular processes closely implicated in tumorigenesis, such as cell proliferation, motility, and angiogenesis [16]. Recent research showed that aberrant expression and activation of PAKs have been observed during the pathogenesis of tumors, causing them accepted as tumor enhancer [17, 18]. However, there is no elucidation about the association between PAK3 and gastric cancer.

In the present study, we validated the expression of circ_0000190 in gastric cancer and found that decreased circRNA expression was associated with poor prognosis of gastric cancer. More importantly, we noted that circ_000190 acted as a sponge of miR-1252 and resultantly increased the expression of PAK3, eventually leading to decrease in cell proliferation and metastasis of gastric cancer cells.

## Materials and methods

### Patients and samples collection

Twenty patients with gastric cancer receiving radical surgery were recruited in this study. The patients had not been treated with any therapies to treat the cancer before the cancer and adjacent normal tissues from patients were collected from December 2012 to May 2015 at The First Affiliated Hospital of Jinzhou Medical University. The samples were immediately frozen in liquid nitrogen and then stored at − 80 °C before use. The study was approved by the Ethics Committee of The First Affiliated Hospital of Jinzhou Medical University and the written informed consents were provided by the recruited patients before using the samples.

### Cell culture and transfection

Human gastric cancer cell lines BGC823 and MGC803 were obtained from Cell Bank of Type Culture Collection (Shanghai, China). The cell lines were cultured using RPMI 1640 medium with 10% fetal bovine serum (FBS; Gibco, NY, USA) at 37 °C and 5% CO_2_. The synthetic mimics of miR-1252 were obtained from GenePharma (Shanghai, China). The sequence of circ_0000190 and PAK3 was obtained by PCR, and then inserted into pcDNA3.0 vectors (Invitrogen, CA, USA) after confirmation. As respective negative controls (NC), the scramble RNA and empty expression vectors were used in this study. The BGC-823 and MGC803 cells were cultured in 6-well plates for 24 h, and transfection was conducted according to the manufacturer’s protocol with lipofectamine 2000 (Invitrogen, CA, USA).

### Cell proliferation assay

The BGC-823 and MGC-82C cells were cultured in 96-well plates (3 × 10^3^ cells/well). After treatment of 24, 48 and 72 h, 10 μL of CCK-8 (Solarbio, Beijing, China) was added into each well and cells were cultured for another 2 h. The absorbance at 450 nm was detected using an ELx808 microplate reader (Bio-Tek, USA). Cell growth was also examined by 5-Ethynyl-20-deoxyuridine (EdU) assay. After culture of indicated time points, EdU solution (Beyotime, Nantong, China) was added into each well and cells were cultured for another 2 h. Then cells were fixed in 4% PFA and nuclei were counter-stained with DAPI (Beyotime).

### Transwell assay

The maintained cells were refreshed with FBS-free medium and then cultured for another 12 h. After harvesting, cells were suspended in serum-free culture medium containing 0.2% BSA at a density of 2 × 10^5^ cells/mL. Then 100 μL of cells were added to the upper chamber pre-coated with Matrigel (BD, CA, USA), while 700 μL complete medium was added to the lower chamber. After 48 h incubation at 37 °C, cells in the upper chamber were cleaned, and the chambers were fixed in 4% paraformaldehyde and stained in 0.1% crystal violet solution to observe the migrated cells in the lower chamber surface.

### Wound-healing assay

The primary or transfected BGC-823 and MGC-803 cells were cells were cultured in 6-well plates (3 × 10^5^ cells/well). After culturing for 48 h, the cell layers were scratched using 100 μL pipette tips and photographed. Then cells were maintained in serum-free RPMI-1640 for 48 h and photographed under an inverted microscope. The wound width was measured using Image J software.

### Terminal deoxyribonucleotidyl transferase (TdT)-mediated dUTP Nick end labeling (TUNEL) assay

TUNEL assay was used to examine apoptosis of cultured gastric cancer cells and gastric cancer tissues. Cells and tissues were fixed in 4% PFA, and tissues were additionally dehydrated with ethanol solution, embedded with paraffin, rehydrated with ethanol solution, and cut into sections of 4 μm thickness. Eventually, apoptosis of the samples were examined using One Step TUNEL Apoptosis Assay Kit (Beyotime) according to the protocol, and the staining activity was detected using a fluorescence microscopy.

### Colony formation

Cells were plated into 6-well plates (2000 cells/well) and cultured for 2 weeks at 37 °C and 5% CO_2_. Then the cells were fixed in 4% PFA and stained in 0.1% crystal violet solution. The colonies were photographed and those with more than 50 cells were counted under an inverted microscope.

### Flow cytometry

Cells were cells were cultured in 6-well plates (3 × 10^5^ cells/well). The cultured cells were harvested and washed with PBS. After incubation in 100 μg/mL propidium iodide (PI) for 30 min at 4 °C, cell cycle was analyzed on cell cycle by flow cytometry. Apoptotic cells were additionally stained with Annexin V-FITC, and examined by flow cytometry. All cell analysis was analyzed using a FACScan instrument (Becton–Dickinson, CA, USA) and CellQuest software (Becton–Dickinson).

### Real time PCR (RT-PCR)

Total RNAs from collected cells and tissues were extracted using Trizol reagent (Invitrogen). After spectrophotometric quantification, cDNAs were synthesized on ABI 7500 Real-Time PCR System (Applied Biosystems, CA, USA) using Primescript RT Reagents (TaKaRa, Dalian, China) according to the manufacturer instruction. With the same PCR system, RT-PCR was conducted in triplicate with the synthesized cDNA and purchased GoTaq^®^ qPCR Master Mix kit (Promega, WI, USA). The primers used in this study are as follows: circ_0000190: 5′-GCCGAGTGGTAACATGGGAG-3′ and 5′-AGCAGAGCAAGTGGAAACCA-3′; GAPDH: 5′-TGTTCGTCATGGGTGTGAAC-3′ and 5′-ATGGCATGGACTGTGGTCAT-3′; miR-1252: 5′-ACACTCCAGCTGGGAGAAGGAAATTGAATTCA-3′ and 5′-CTCAACTGGTGTCGTGGAGTCGGCAATTCAGTTGAGTAAATGAA-3′; U6: 5′-CTCGCTTCGGCAGCACA-3′ and 5′-AACGCTTCACGAATTTGCGT-3′. The reaction process initiated with a denaturation at 94 °C for 30 s, and then included 40 cycles of 30 s denaturation at 94 °C, 20 s anneal at 60 °C, and 20 s elongation at 68 °C. GAPHD and U6 were employed as internal control to calculate the relative level of interested genes (2^−ΔΔCT^).

### Western blot

Cells were cells were cultured in 6-well plates (3 × 10^5^ cells/well). The harvested cultured cells were lysed and the frozen cancer tissues were homogenized using RIPA lysis buffer (Beyotime, Haimen, China). The cell lysates and homogenates were centrifuged for 10 min at 8000*g* and 4 °C followed by collection of the supernatants. After protein concentration quantification using BCA assay (Bio-Rad, CA, USA) and subsequent denaturation at 95 °C for 5 min, the samples were loaded onto 10% sodium dodecyl sulfate–polyacrylamide gels (SDS-PAGE), subjected to electrophoresis and transfer to polyvinylidene difluoride (PVDF) membrane (Bio-Rad). After blocking with 5% bovine serum albumin (BSA) at room temperature for 1 h, the blots were incubated overnight at 4 °C with primary antibodies against PAK3, caspase-3, cyclin D, p27 and GAPDH (Santa Cruz, CA, USA), and then incubated with HRP-conjugated secondary antibody for 1 h. The immuno-reactivity was determined by ECL detection reagents (GE healthcare, USA).

### Immunohistochemistry (IHC)

The collected xenograft tumor tissues from mice were cut into sections as above mentioned. The sections were incubated with the primary antibody of Ki-67 (Santa Cruz) at room temperature for 1 h, and then incubated with the secondary antibody. Immunoreactivity was visualized using diaminobenzidine (DAB) kit (Beyotime) and photographed under a microscope (Olympus, Japan).

### Fluorescence in situ hybridization (FISH)

Cells were cultured on coverslips to exponential phase and then fixed in 4% PFA. The 4 μm sections of gastric cancer tissues fixed in 4% PFA were prepared as above mentioned. The samples received permeabilization with 0.25% Triton X-100 and hybridization in buffer containing biotin-conjugated probes (GenePharma) against circ_0000190. After blocking with 10% normal goat serum in PBS, the samples were incubated with FITC-streptavidin (Invitrogen) overnight at 4 °C. After incubation, cells were washed with TBS, and then counter-stained with DAPI.

### Dual-luciferase reporter assays

Circular RNA Interactome (https://circinteractome.nia.nih.gov) and TargetScan (http://www.targetscan.org) were employed to predict the interaction between Circ_0000190, miR-1252 and PAK3. Dual-luciferase reporter assays were used to detect the binding interactions. In brief, the wild sequences of circ_0000190 and PAK3 3′UTR and the corresponding mutants (MUT) were inserted into PsiCHECK-2-Report vectors (Promega), which were subsequently transfected into MCG823 cells using Lipofectamine 2000 (Invitrogen). Then co-transfection with either miR-1252 mimics or the NC was conducted. After 48 h transfection, the signals were detected and the luciferase activities were calculated by firefly luciferase intensity against renilla’s.

### Athymic nude model experiment

4–6 weeks old of male BALB/c nude mice were obtained from Guangdong Medical Laboratory Animal Center (Guangzhou, China). Mice were housed under constant temperature (20–26 °C) and relative humidity (40–75%), with a cycle of 12 h light/12 h dark. After an acclimatization of at least 5 days, the mice were subjected to subcutaneous injection at the left flank of 1 × 10^7^ of BGC-823 cells, which had been transfected with Lv-circ_0000190 or NC and individually responded. Every 7 days, the tumor volume was measured to calculate the size by the formula: 0.5 × length × width^2^. After 28 days, the mice were sacrificed and the tumor tissues were collected. A part was stored at − 80 °C and the other stored in 4% PFA before use. The experiment was approved by the Ethics Committee of The First Affiliated Hospital of Jinzhou Medical University.

### Statistical analysis

All experiments were conducted for at least three independent times, and the data were analyzed by SPSS 19.0 (SPSS, IL, USA) and expressed as mean ± SEM. Student’s *t* test was used to for comparisons of two groups, while one-way analysis of variance (ANOVA) followed by Duncan’s test was conducted to evaluate the difference among multiple groups. *P* < 0.05 was considered statistically significant.

## Results

### Circ_0000190 is decreased in gastric cancers

**Fig. 1:**
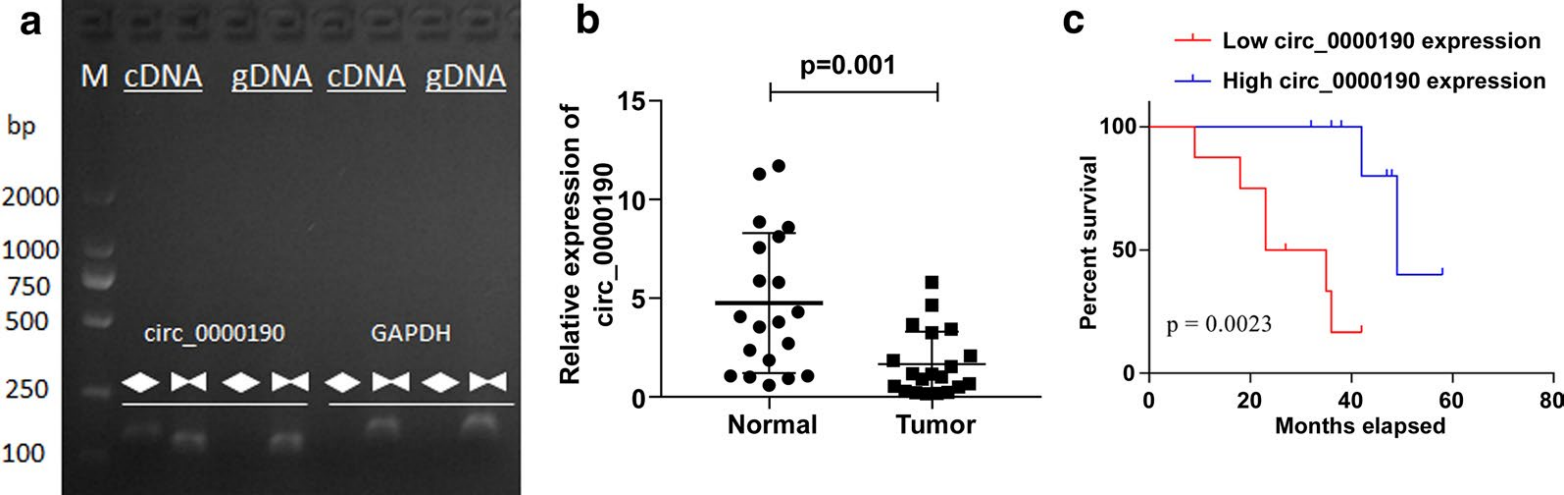
Circ_0000190 expression was down-regulated in gastric cancer. **a** cDNA and gDNA were used to detect circ_0000190. **b** Circ_0000190 expression in human cancer cells and normal tissues were detected by RT-PCR. **c** Overall survival curve analysis in gastric cancer patients with low or high expression of circ_0000190

We firstly confirmed circ_0000190 via PCR. As shown in Fig. 1a, PCR with cDNA yielded a product with sequence consistent with circ_0000190, while no products were obtained in gDNA. The examination of the circRNA expressions showed a significant decrease in gastric cancer tissues compared with the peritumor normal tissues (Fig. 1b), which indicated that circ_0000190 might participate in the progress of gastric cancers. Furthermore, a survival curve analysis was made to observe the prognostic information of circ_0000190 in patients with gastric cancers. Interestingly, a high expression of the circRNA was accompanied with an increase in the risk of survival, while a lower expression with a higher overall survival (Fig. 1c). These findings suggested that circ_0000190 might be a potential prognosis biomarker of gastric cancers.

### Circ_0000190 inhibits gastric cancer cell growth

**Fig. 2:**
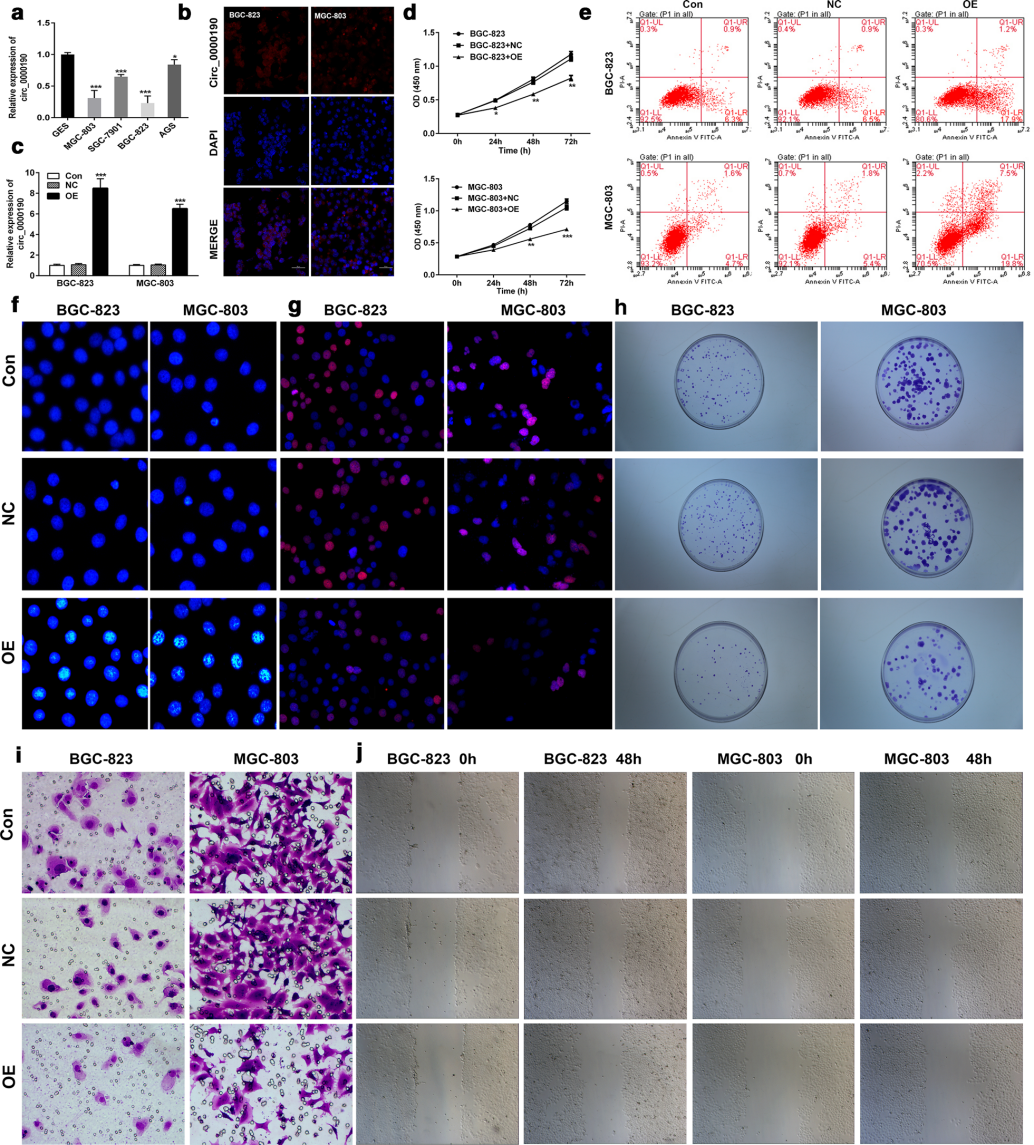
Circ_0000190 inhibited cancer cell growth. **a** Expression level of circ_0000190 in normal gastric cells (GES) and gastric cancer cells (MGC-803, SGC-7901, BGC-823 and AGS) was detected by RT-PCR, *P < 0.05, ***P < 0.001 compared with GEC cells. **b** Location of circ_0000190 in BGC-823 and MGC-803 cells was examined by FISH. The empty vectors and those inserted with circ_0000190 were individually transfected into BGC-823 and MGC-803 cells. **c** The expressions of circ_0000190 in the primary or transfected BGC-823 and MGC803 cells was examined by RT-PCR, ***P < 0.001 compared with the control or NC cells. **d** Cell viability were examined by CCK-8 assay at 24 h, 48 h and 72 h after culture, ***P < 0.001 compared with the control or NC cells at the same condition. Cell apoptosis of the primary and transfected BGC-823 and MGC-803 cells was examined by flow cytometry (**e**) and Hoechst staining (**f**), the cell proliferation was detected by EdU (**g**) and colony formation assay (**h**), cell invasion and migration were examined by transwell (**i**) and scratch (**j**) assay, respectively

To investigate the role of circ_0000190 in gastric cancers, the expression of circ_0000190 was determined in multiple gastric cancer cell lines firstly. Our results showed that the circRNA levels were significantly decreased in those detected gastric cancer cell lines, including MGC-803, SGC-7901, BGC-823 and AGS, compared with the normal gastric epithelial cell line GEC (Fig. 2a). As circ_0000190 expression in MGC-803 and BGC-823 cells were much lower than other gastric epithelial cell lines, those cell lines were employed for the following studies. Subsequently, the expression of the circRNA was additionally confirmed by FISH assay (Fig. 2b). In order to evaluate the function of circ_0000190 in gastric cancer cells, we successfully established circ_0000190 overexpression cell lines (Fig. 2c). Interestingly, overexpression of circ_0000190 was accompanied with a significant decrease in cell viability, which emerged at the onset of 24 h and sustained until 72 h after transfection (Fig. 2d). In addition, flow cytometry analysis showed that apoptosis of the cells with circ_0000190 overexpression was significantly increased (Fig. 2e), which was consistent with the result of Hoechst staining (Fig. 2f). Moreover, the cell proliferation examination by Edu and colony formation assay suggested that circ_0000190 overexpression significantly suppressed the number of cells and colonies (Fig. 2g, h). Eepithelial mesenchymal transition (EMT) is considered as an evolutionarily conserved developmental process associated with carcinogenesis. So we detected the effect of circ_0000190 overexpression on EMT by observing cell migration/invasion. Our results demonstrated that circ_0000190 overexpression resulted in a decrease of cell migration/invasion, suggesting that circ_0000190 overexpression inhibited EMT (Fig. 2i, j). All these results indicated that circ_0000190 acts as an anti-oncogenic regulator in gastric cancers.

### Circ_0000190 regulates the cell cycle of gastric cancer cells

**Fig. 3:**
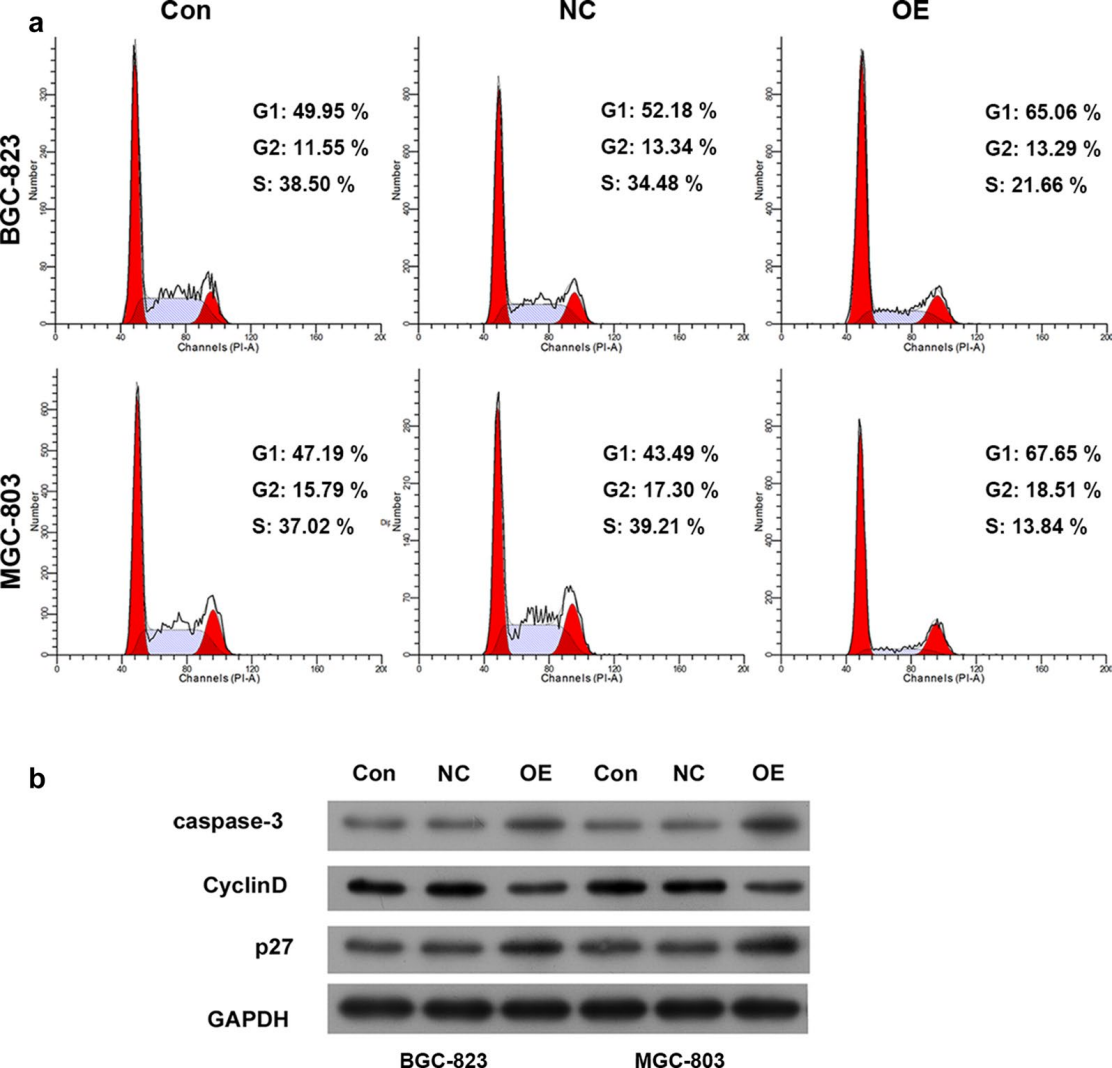
Circ_0000190 induced cell cycle arrest. Cell cycle of The primary gastric cancer cells and transfected cells were observed by flow cytometry (**a**), and the expressions of caspase-3, CyclinD and p27 were examined by Western blot (**b**)

Because cell proliferation could be affected through altering cell cycle. So we determined the effect of circ_0000190 overexpression on cell cycle of gastric cancer cells by flow cytometry. As shown in Fig. 3a, circ_0000190 overexpression significantly increased the cell population at G1 phase in both BGC-823 and MGC-803 cells. Concurrently, a decrease in S phase was noted in gastric cancer cells with circ_0000190 overexpression. These results were indicating the suppressive effect by circ_0000190 on DNA synthesis in gastric cancer cells. In addition, Western blot assay showed that caspase-3 and p27 levels were significantly enhanced, while cyclin D level was decreased after circ_0000190 overexpression (Fig. 3b), which suggested that circ_0000190 regulates the expression of p27 and cyclinD, and resultantly regulated the cell cycle in gastric cancer cells.

### MiR-1252 is a target of circ_0000190 and directly targeting PAK3

**Fig. 4:**
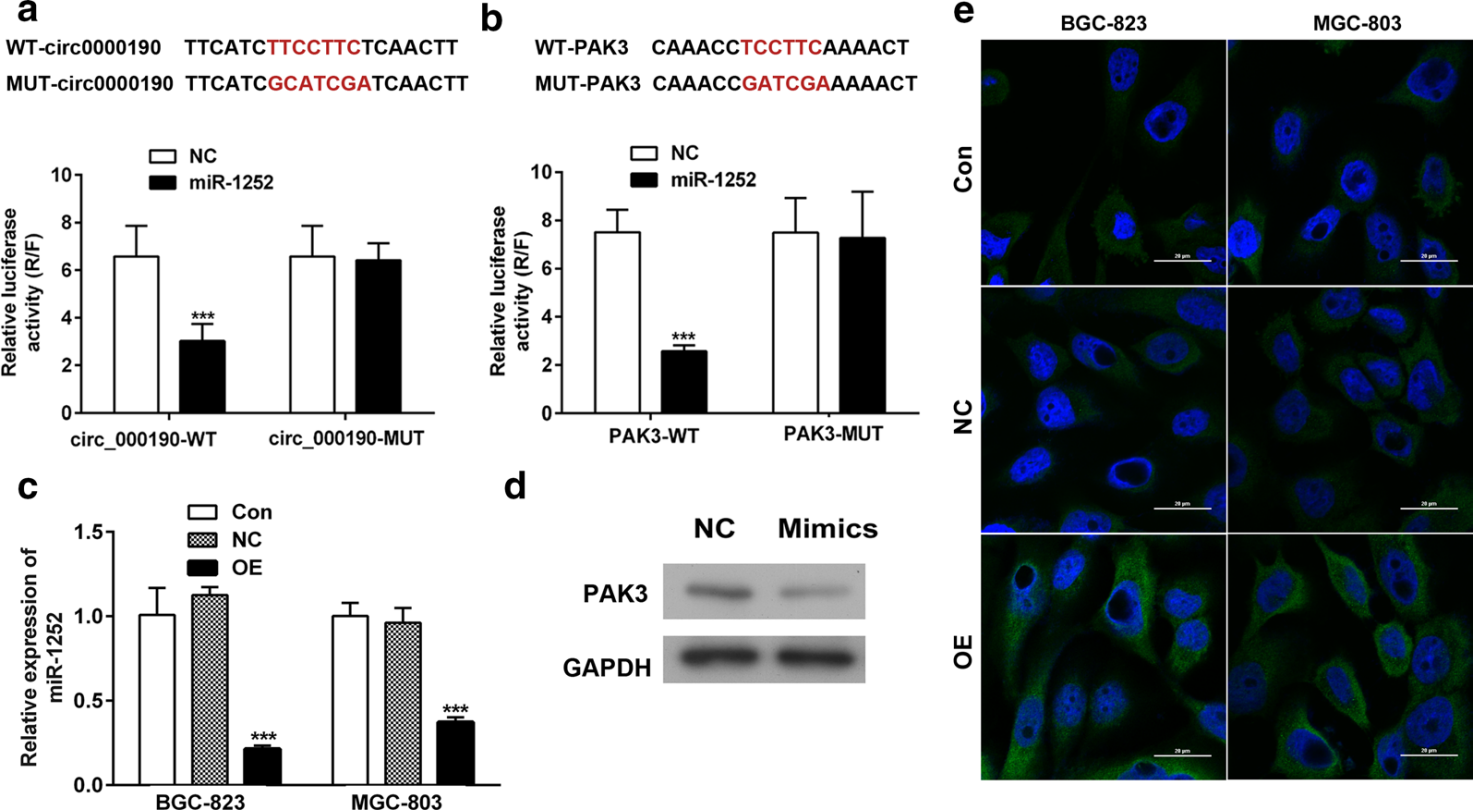
MiR-1252 is a sponge of circ_0000190 and directly targeted PAK3. The binding of miR-1252 between circ_0000190 (**a**) and PAK3 (**b**) were predicted, and then examined by luciferase reporter assay. ***P < 0.001, compared with NC. The expressions of miR-1252 in the primary or/and transfected cells were detected by RT-PCR (**c**), and PAK3 levels were detected by Western blot (**d**) and immunofluorescence assay (**e**) (blue DAPI, green PAK3). ***P < 0.001, compared with NC or control

Because the functions of circRNAs associated with various biological process were majorly ascribed to sponging microRNAs. Therefore, we are wondering to found the most related microRNAs of circ_0000190. According to the starbase, circ_0000190 was predicted as a sponge of miR-1252, and the potential binding sites were also identified. Using another database, TargetScan, we identified PAK3 as a direct target of miR-1252, and the potential binding sites (Fig. 4a, b). As expected, the luciferase reporter assay showed that overexpression of miR-1252 significantly suppressed the luciferase activity of reporter genes with wide type of either PAK3 or circ_0000190, and had no effect on those of the relevant MUT reporters (Fig. 4a, b), indicating that circ_0000190 directly targeted miR-1252, which in turn directly targeted PAK3. Consistently, we also found that circ_0000190 overexpression significantly decreased miR-1252 level and increased PAK3 expression, while miR-1252 mimics significantly decreased PAK3 expression in gastric cancer cells (Fig. 4c–e).

### MiR-1252 is increased and PAK3 is decreased in gastric cancers

**Fig. 5:**
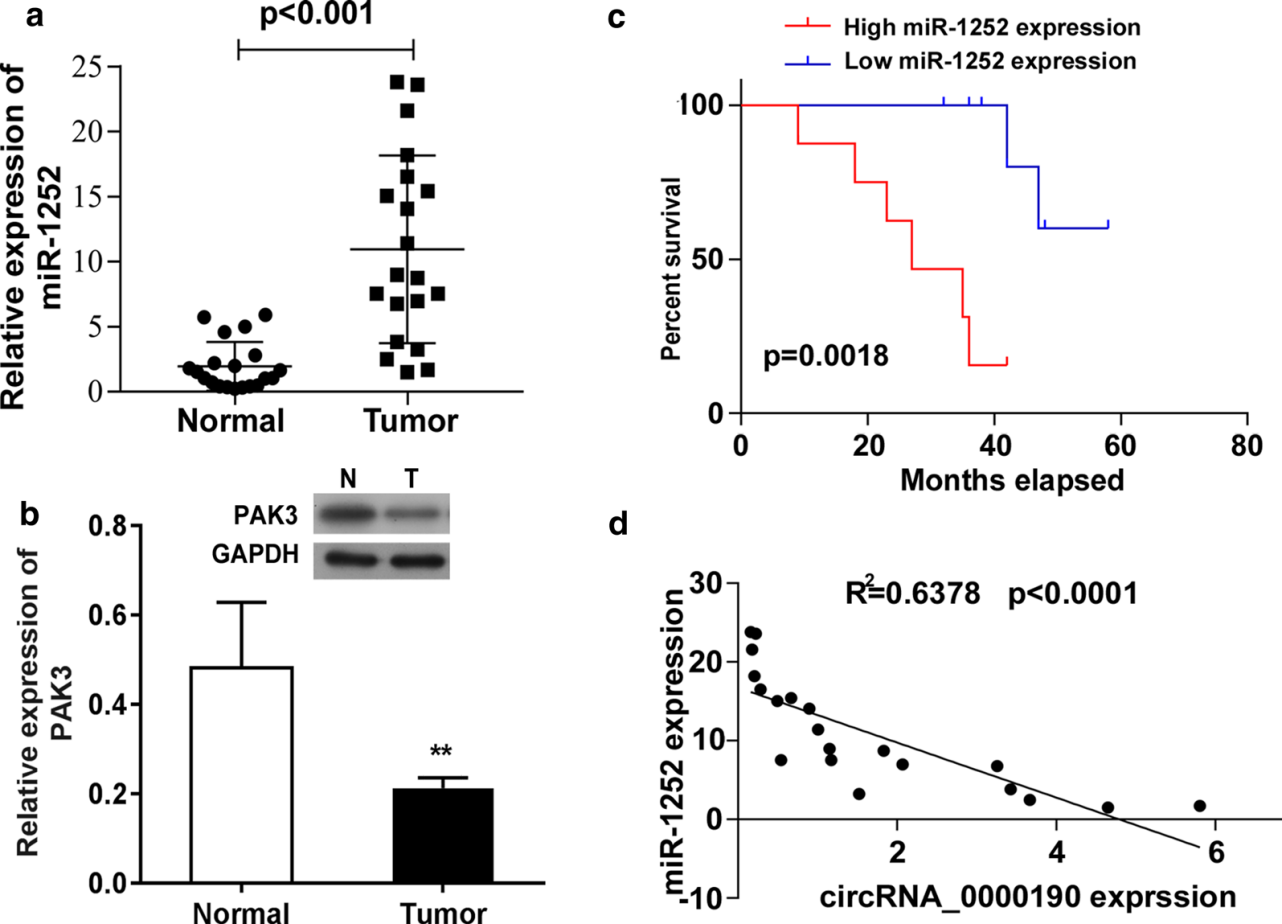
MiR-1252 is increased and PAK3 is decreased in gastric cancer. The expression of miR-1252 and PAK3 were determined by RT-PCR (**a**) and Western blot (**b**). **P < 0.01, compared with normal tissue. **c** The overall survival of gastric cancer patients with high or low miR-1252 expression was analyzed. **d** The correlation between miR-1252 expression and circ_0000190 expression in gastric cancer was analyzed

Next, we further explored the relation between miR-1252 and PAK3 in gastric cancer. As shown in Fig. 5a, b, miR-1252 and PAK3 were significantly increased and decreased in tumor tissues, respectively. In addition, the prognostic evaluation also showed that the survival rate in miR-1252 high expression group was significant better than that in low expression group (Fig. 5c). Furthermore, we also found that the expression correlation analysis between miR-1252 and circ_0000190 exhibited a negative correlationship (Fig. 5d). These data indicated that the miR-1252 might participated in the progress of gastric cancer, which might be associated with PAK3.

### Circ_0000190/miR-1525/PAK3 pathway on the cell progression of gastric cancer

**Fig. 6:**
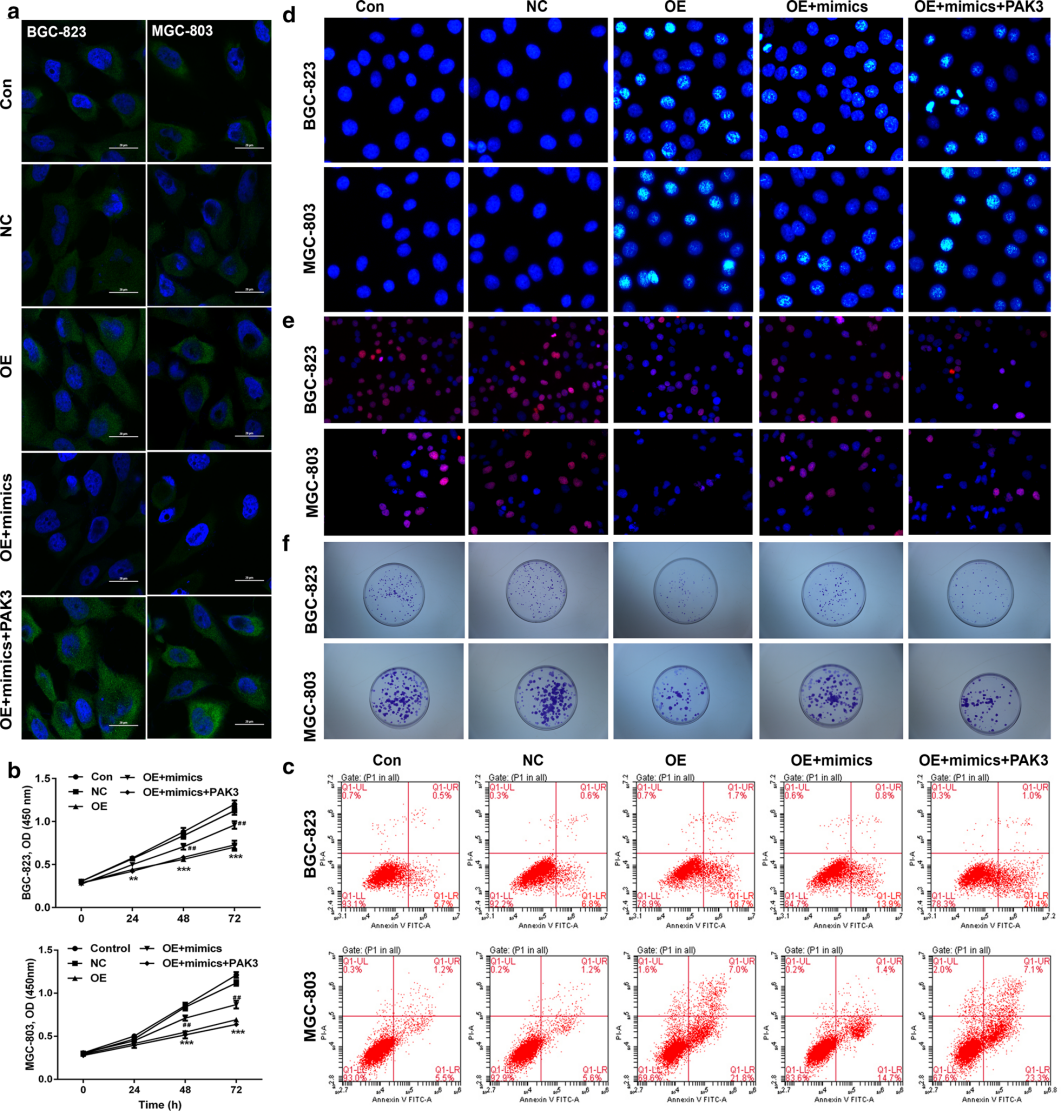
MiR-1252/PAK3 regulated inhibitory effect by circ_0000190 on gastric cancer cell growth. BGC-823 and MGC-803 cells were transfected with empty vectors or vectors inserted with circ_0000190 alone, or in combination with miR-1252 mimics or miR-1252 mimics and vectors containing PAK3 cDNA. PAK3 expression was determined by immunofluorescence (**a**) (blue DAPI, green PAK3). Cell viability was examined by CCK-8 assay, **P < 0.01, ***P < 0.001, compared with NC; ^##^*P *< 0.01, compared with OE andOE + mimics + PAK3 (**b**), Apoptosis was assayed by flow cytometry (**c**) and Hoechst staining (**d**). The cell proliferation was determined by Edu (**e**) and colony formation assay (**f**)

**Fig. 7:**
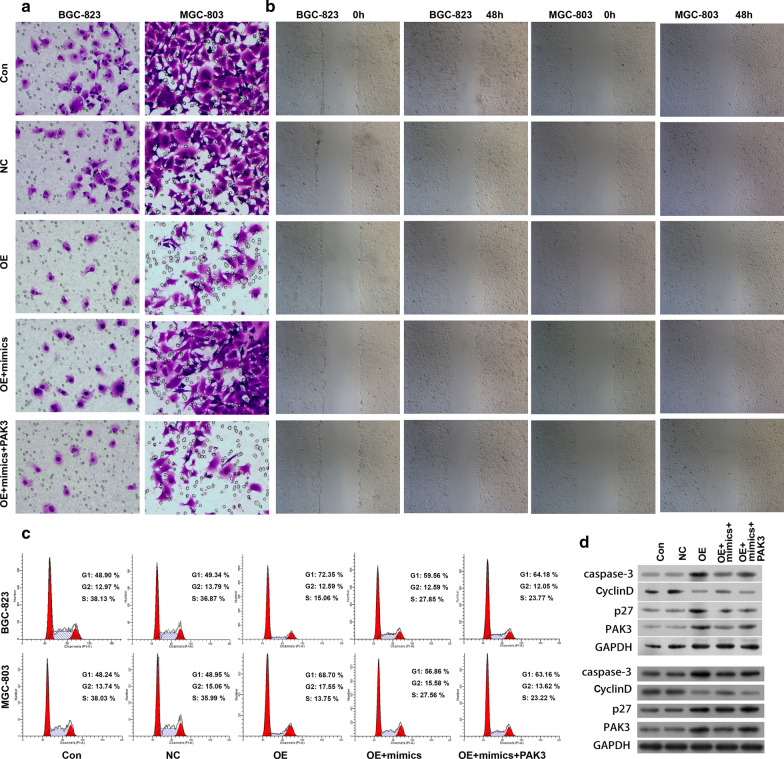
MiR-1252/PAK3 regulated inhibitory effect by circ_0000190 on gastric cancer migration and cell cycle. Cell invasion and migration was assayed by transwell (**a**) and scratch (**b**), respectively. Cell cycle was detected by flow cytometry (**c**), and the expression of caspase-3, CyclinD, p27 and PAK3 was determined by Western blot (**d**)

Base on the findings we described above, we are curious to know the correlation between circ_0000190, miR-1252 and PAK3. The immunofluorescence staining showed that PAK3 expression was significantly inhibited after miR-1252 mimics transfection, which could be reversed after additional transfection with PAK3 in gastric cancer cells (Fig. 6a). Subsequently, cell growth were determined. We found that the cell viability was significantly increased by circ_0000190, and inhibited by addition of miR-1252 mimics, which could be abolished by further overexpression of PAK3 in gastric cancer cells (Fig. 6b). Consistently, circ_0000190 overexpression increased apoptosis (Fig. 6c, d), decreased cell proliferation (Fig. 6e, f) and migration (Fig. 7a, b) of gastric cancer cells, which were inhibited after miR-1252 mimics transfection and then restored by further transfection with PAK3. These findings suggested that miR-1252/PAK3 mediated the anti-proliferation by circ_0000190. In addition, cell cycle analysis also supported that circ_0000190 overexpression blocked cell cycle at G1 phase and suppressed cell cycle (Fig. 7c), which attenuated by additional transfection of miR-1252 mimics, while PAK3 overexpression rescued the cell cycle arrest. Moreover, We also found that the pro-apoptotic protein, caspase-3 was significantly increased by circ_0000190 transfection, and then suppressed by co-transfection of mimiR-1252, but restored by overexpression of PAK3 in gastric cancer cells (Fig. 7d). Contrary and similar effects were found in the expression of Cyclin D and p27 in detected gastric cancer cells, respectively (Fig. 7d). These results suggested that circ_0000190/miR-1252/PAK3 axis regulated the cell progression of gastric cancer cells.

### Circ_0000190 retarded human gastric tumor growth in mice xenograft model

**Fig. 8:**
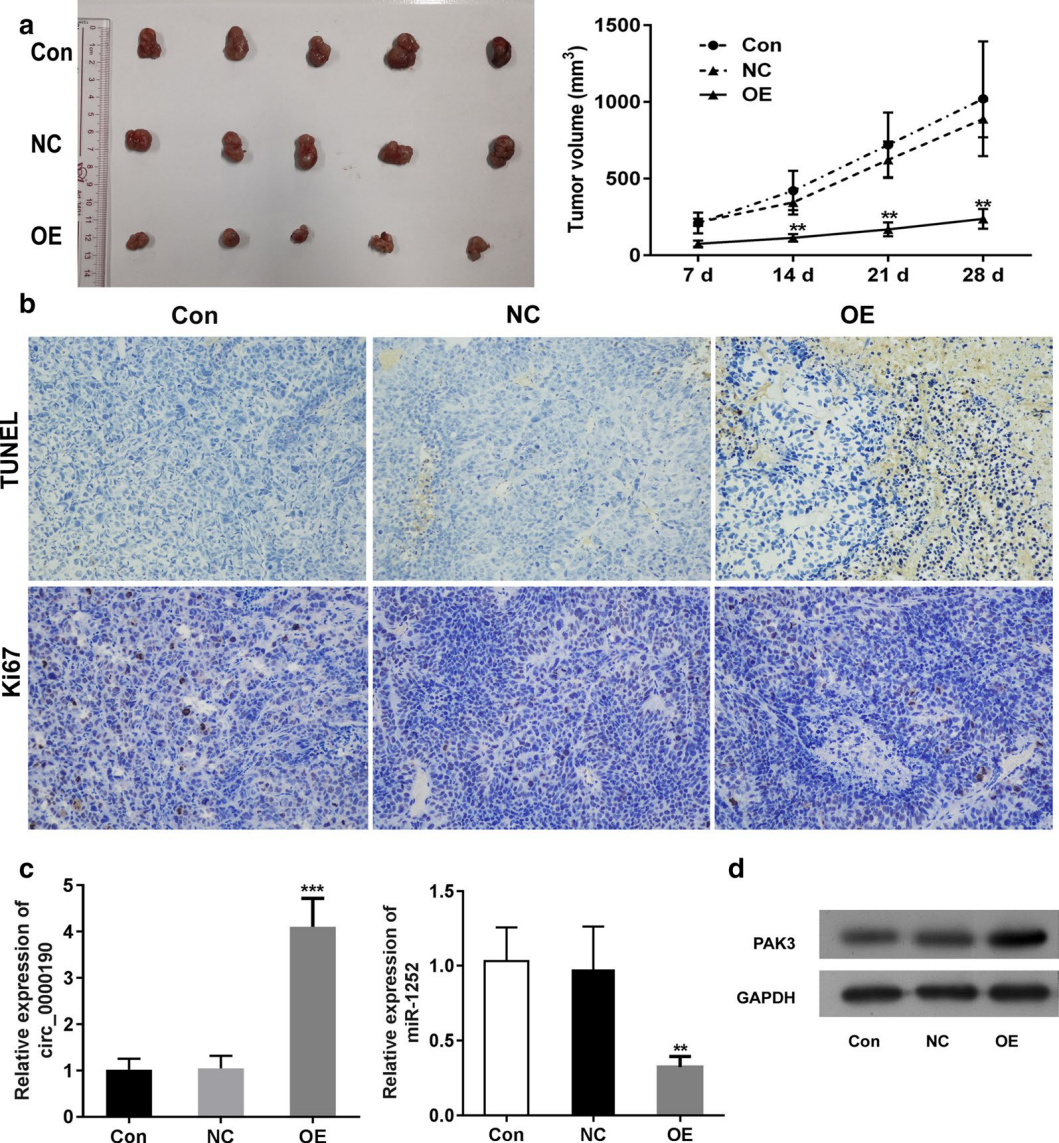
Circ_0000190 inhibited human gastric cancer growth in xenograft mice model. The primary BGC-823 cells, BGC-823 cells transfected with circ_0000190 or NC were subcutaneously injected at the left flank. The growth curve and the size of xenograft tumors after 28 days were determined. The tumor volumes were detected weekly after seeding (**a**) Apoptosis and cell proliferation were determined by TUNEL assay. **b** Ki67 expression was detected by ICH. The expressions of circ_0000190, miR-1252 (**c**), and PAK3 (**d**) were detected by RT-PCR and Western blot, respectively

To evaluate the potential effect by circ_0000190 in gastric cancer growth in vivo, mice xenograft model were established with primary BGC-823 cells or BGC-823 cells with circ_0000190 overexpression. As shown in Fig. 8a, circ_0000190 overexpression significantly retarded tumor xenograft. Consistently, significant increase in apoptotic cells and significant decrease in ki67 positive cells were observed in circ_0000190 overexpression group (Fig. 8b). We also found that circ_0000190 overexpression led to decrease in miR-132 level and increase in PAK3 protein expression of tumor xenograft (Fig. 8c, d). These results supported the antitumoral effect of circ_0000190 on human gastric cancer by regulating miR-1252/PAK3 axis.

## Discussion

Gastric cancer remains the fifth most common cancer and third leading cause of cancer death all over the world. Despite intensive effort has been made to treat gastric cancer, gastric cancer still results in a 5-year overall survival less than 30%. Therefore, it is an urgent need to develop a novel therapy against gastric cancer.

Emerging studies have noted aberrant expression of circRNAs and their correlation in tumor progression [19]. Further investigation revealed that circRNAs play an important role in the biological behavior of gastric cancer cells. For example, circ_104916 expression was down-regulated in gastric cancer tissues and cells, functioning as a tumor inhibitor by regulating the proliferation, migration, invasion and EMT of gastric cancer cells [20]. CircLARP4 was found has a lower level in gastric cancer tissues than adjacent normal gastric tissues, and demonstrated to suppress gastric cancer development [21]. Likewise, decrease of circ_0000190 expression has been reported in gastric cancer [10]. Moreover, in multiple myeloma patients, circ_0000190 was also found down-regulated in bone marrow and peripheral blood [15]. Interestingly, it has been noted that there is a close correlation bewteen circ_0000190 expression and gastric cancer size, lymphatic metastasis, distal metastasis as well as tumor-node-metastasis. It suggested that circ_0000190 expression exhibits higher sensitivity and specificity in screening gastric cancer, as compared with the most commonly used serum gastric cancer markers, CEA and CA19-9 [10]. In the current study, we found circ_0000190 expression was associated with the overall survival of gastric cancer patients. We also confirmed that circ_0000190 inhibited gastric cancer cell viability, proliferation and migration, and induced apoptosis and cell cycle arrest. Moreover, we demonstrated that overexpression of circ_0000190 effectively inhibited the growth of gastric cancer xenograft. These findings implicated that circ_0000190 might be a promising therapeutic strategy against gastric cancer.

Because the functions of circRNAs associated with various biological process were majorly ascribed to sponging microRNAs. To study the underlying mechanism how circ_0000190 regulating gastric cancer progression, we found that the circRNA was validated as a sponge of miR-1252. It is ubiquitous that a circRNA could directly sponge several miRNAs, such as circPTPRA, which is a sponge of miR-636 and miR-96-5p [22, 23]. Reasonably and acceptably, the functions of a circRNA could be simultaneously and negatively regulated by several miRNAs. In this study, we revealed that miR-1252 mimics only partially blocked the inhibitory effect by circ_0000190 on cell proliferation. Therefore, the role of circ_0000190 in gastric cancer might also be regulated another miRNA, such as miR-767-5p, which need to be further investigated.

**Fig. 9:**
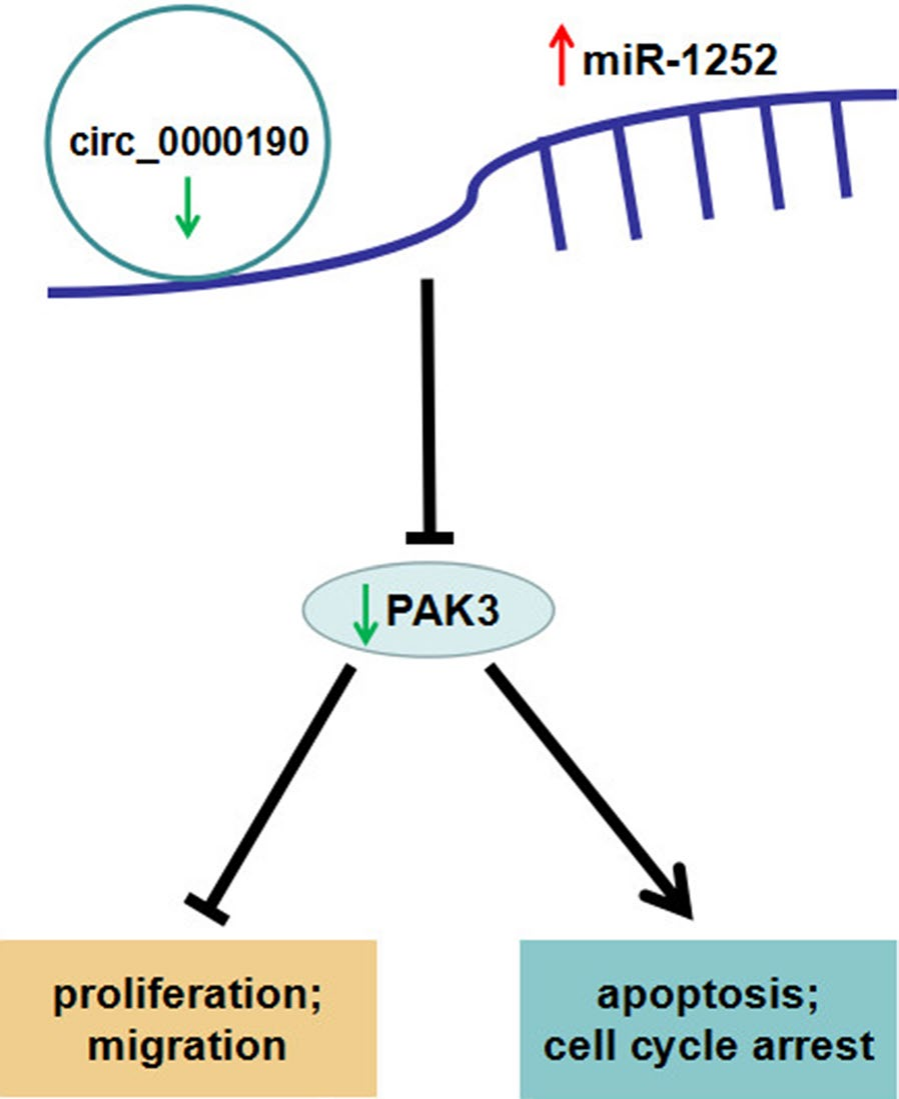
Schematic diagram of circ_0000190 suppresses gastric cancer progression

PAK is a serine/threonine kinase that play an important role in a variety of cellular processes closely implicated in tumorigenesis. Recent research reported that aberrant expression and activation of PAKs have been found during the pathogenesis of tumors, causing them accepted as tumor enhancer. In this study, we found that PAK3 expression was aberrantly down-regulated in gastric cancer, and PAK3 participated in gastric cancer cell cycle arrest, apoptosis, cell proliferation and migration. To further reveal the mechanism how circ_0000190 suppresses gastric cancer progression, we discovered that the effects of circ_0000190 on the cellular processes such as proliferation, migration, apoptosis and cell cycle arrest in gastric cancer were blocked by miR-1252 mimics, which could be rescued after further overexpression of PAK3. In summary, these findings supported that circ_0000190 suppresses gastric cancer progression potentially directly targeted miR-1252/PAK pathway (Fig. 9).

## Conclusion

In conclusion, we demonstrated that circ_0000190 is closely associated with gastric cancer progression. Functional and mechanistic analysis supported that circ_0000190 overexpression markedly decreased gastric cancer cell survival, growth and migration, which was achieved by directly targeting miR-1252/PAK3 axis. Overall, we revealed that circ_0000190/miR-1252/PAK3 may serve as a potential novel strategy for the treatment of gastric cancer.

## Data Availability

All data generated or analysed during this study are included in this published article.

## Abbreviations

CircRNAs: Circular RNAs
miRNAs: MicroRNAs
PAKs: P21-activated kinases
NC: Negative controls
EdU: 5-Ethynyl-20-deoxyuridine
TdT: Terminal deoxyribonucleotidyl Transferase
PI: Propidium iodide
RT-PCR: Real time PCR
SDS-PAGE: Sodium dodecyl sulfate–polyacrylamide gels
PVDF: Polyvinylidene difluoride
BSA: Bovine serum albumin
IHC: Immunohistochemistry
DAB: Diaminobenzidine
FISH: Fluorescence in situ hybridization
ANOVA: Analysis of variance
